# Identification of Allelic Heterogeneity at Type-2 Diabetes Loci and Impact on Prediction

**DOI:** 10.1371/journal.pone.0113072

**Published:** 2014-11-13

**Authors:** Yann C. Klimentidis, Jin Zhou, Nathan E. Wineinger

**Affiliations:** 1 Mel and Enid Zuckerman College of Public Health, Division of Epidemiology and Biostatistics, University of Arizona, Tucson, Arizona, United States of America; 2 Scripps Translational Science Institute, La Jolla, California, United States of America; Tulane School of Public Health and Tropical Medicine, United States of America

## Abstract

Although over 60 single nucleotide polymorphisms (SNPs) have been identified by meta-analysis of genome-wide association studies for type-2 diabetes (T2D) among individuals of European descent, much of the genetic variation remains unexplained. There are likely many more SNPs that contribute to variation in T2D risk, some of which may lie in the regions surrounding established SNPs - a phenomenon often referred to as allelic heterogeneity. Here, we use the summary statistics from the DIAGRAM consortium meta-analysis of T2D genome-wide association studies along with linkage disequilibrium patterns inferred from a large reference sample to identify novel SNPs associated with T2D surrounding each of the previously established risk loci. We then examine the extent to which the use of these additional SNPs improves prediction of T2D risk in an independent validation dataset. Our results suggest that multiple SNPs at each of 3 loci contribute to T2D susceptibility (*TCF7L2*, *CDKN2A/B*, and *KCNQ1*; p<5×10^−8^). Using a less stringent threshold (p<5×10^−4^), we identify 34 additional loci with multiple associated SNPs. The addition of these SNPs slightly improves T2D prediction compared to the use of only the respective lead SNPs, when assessed using an independent validation cohort. Our findings suggest that some currently established T2D risk loci likely harbor multiple polymorphisms which contribute independently and collectively to T2D risk. This opens a promising avenue for improving prediction of T2D, and for a better understanding of the genetic architecture of T2D.

## Introduction

Approximately 65 loci have been shown to be associated with type-2 diabetes (T2D) through genome-wide association studies (GWAS). However, variation at these loci accounts for a small proportion of the expected heritability of T2D [1], [2]. Among several potential strategies for identifying additional contributing genetic variation, one approach is to determine whether there are additional genetic markers near established loci that act independently or jointly with the reported marker (lead SNP).

Allelic heterogeneity is a feature of the genetic architecture of many traits, including common traits and diseases such as height, BMI, and T2D [3]–[6]. In the context of T2D genetics, both Morris et al. [2] and Yang et al. [7] have suggested that additional SNPs in established loci are associated with T2D risk. However, Morris et al. only considered SNPs in weak linkage disequilibrium (r^2^<0.05) with the lead SNP, and that were not in the same recombination interval. Hence, without formal conditional analysis, they identified two loci as having multiple associations at genome-wide significance (*KCNQ1* and *CDKN2A/B*), and two more at suggestive levels (*DGKB* and *MC4R*). Yang et al. have recently developed a method for identifying additional associated SNPs based on conditional/joint (C/J) analysis using GWAS summary statistics and linkage disequilibrium (LD) information from a reference sample [7]. They applied their method to only a single established T2D locus (*CDKN2A/B*), and identified two novel SNPs at that locus that were significantly associated with T2D when fitted jointly. Finally, on a smaller scale (1,924 cases and 5,380 controls), Ke [8] identified multiple associated loci at the *CDKN2A/B* and *TSPAN8* loci. Although higher power is afforded with the GWAS meta-analysis approach to identify associations with single SNPs, it does not allow for direct C/J analysis since the actual genotype data is not available. The advantage of the method developed by Yang et al. is that it takes advantage of the greater power of GWAS meta-analyses, while also testing for C/J associations, which would otherwise be impossible without individual level data.

Here, we comprehensively examine allelic heterogeneity based on the method developed by Yang et al. at 65 T2D loci discovered by the DIAGRAM consortium, using the summary statistics from their recent meta-analysis of T2D GWAS. We then examine the extent to which these newly identified SNPs increase the accuracy of T2D risk prediction in an independent validation dataset.

## Methods

### Datasets

We used 6,054 nominally unrelated European-American subjects (genomic relationship coefficient <0.025, based on approximately 2.5 million SNPs) from the Atherosclerosis Risk in Communities (ARIC) study [9] to obtain linkage disequilibrium (LD) estimates. According to Yang et al. [7], this sample size is sufficient for LD estimation with minimal error. In order to maximize the overlap of SNPs between the meta-analysis summary statistics (see below) and the ARIC study, we used IMPUTE2 software [10] along with 1000 Genomes reference data to impute millions of additional SNPs. Prior to imputation, we excluded individuals with a high genotype missing rate (>10%). SNPs were excluded based on extreme minor allele frequency (<0.5%), a high missing rate (>10%), or failed Hardy-Weinberg equilibrium (p<0.005). After imputation, we excluded SNPs with ‘info’ <0.6 (measure of imputation quality), and SNPs with genotype dosage between 0.33 and 0.66, or between 1.33 and 1.66. Intermediate dosages outside of these specified ranges were rounded to the nearest integer. We did not use intermediate genotype dosages since this was not an option with the GCTA software, described below.

The validation dataset consisted of European-American subjects from the Multi-Ethnic Study of Atherosclerosis (MESA) [11], which included 225 T2D cases and 1,985 controls. T2D cases were defined as having a fasting glucose level ≥126 mg/dL, a self-report of taking diabetes medication, or a physician diagnosis of T2D. This dataset can be considered an independent validation dataset since it was not part of the DIAGRAM meta-analysis, whereas ARIC was a part of this meta-analysis, thus precluding it from any validation assessment. We implemented genotype QC and imputation as detailed above. However, we did not round or remove intermediate genotype dosages. The MESA and ARIC dataset were obtained from dbGaP (database of Genotypes and Phenotypes). IRB approval was obtained from the University of Arizona.

### Conditional/Joint Analysis

Using the summary statistics from the discovery phase of the latest version (v3) of the DIAbetes Genetics Replication And Meta-analysis (DIAGRAM) consortium, available to the public through an online source [12] and LD estimates from the ARIC dataset as described above, we used GCTA software [13] to perform stepwise model selection. Briefly, SNPs were selected into the model based on p-values in the meta-analysis. An iterative scheme was adopted in which C/J analyses were alternatively performed with the stepwise selection procedure. SNPs with a re-estimated (i.e. through C/J estimation as opposed to marginal estimation) p-value under a certain threshold were selected. For a full description of the method, see Yang et al. [7]. We restricted our analysis to only the genomic regions within 1 Mb of the top SNP at the 65 established T2D loci as reported in Morris et al [2]. This filtering along with the QC filtering described above resulted in 112,329 SNPs being used in this analysis. For each SNP, we recorded the following information as input for the C/J analysis: effect allele, effect size (log of odds ratio), corresponding standard error, p-value, allele frequency of the effect allele (based on ARIC sample described above, as this was not available in the DIAGRAM summary statistic file), and sample size (sum of cases and controls). We used PolyPhen-2 [14] to determine whether any of the newly identified SNPs had any predicted functional effect, and RegulomeDB [15] to determine whether these SNPs may lie in regulatory regions (e.g. transcription factor binding sites) or are associated with specific DNA features (e.g. DNAse sensitivity).

### Validation/Prediction

We compared several prediction models. First we constructed a baseline model which only considered demographic information (sex and age). Then we added a weighted genetic risk score [16] based on only the set of lead SNPs with weights corresponding to the log odds ratios according to the DIAGRAM meta-analysis summary statistics. Lead SNPs were defined as those that had the lowest p-value in the respective 2 Mb region according to the DIAGRAM Stage 1 meta-analysis summary statistics. We then considered a weighted genetic risk score based on all SNPs identified by the C/J analysis with weights corresponding to the coefficients estimated from the C/J analysis. We conducted the above analyses at the following p-value thresholds based on the C/J results: 5×10^−8^, 5×10^−7^, 5×10^−6^, 5×10^−5^, and 5×10^−4^. We examined the proportion of variance explained by these additional SNPs by calculating the variance explained on the liability scale, estimated through the odds ratios and allele frequencies of the SNPs, and assuming a disease prevalence of 10%, using the Mangrove package [17] in R [18]. We also calculated Nagelkerke's R^2^ [19] of the models which include age and sex and each of the GRS, using the fmsb package [20] in R, and report the Akaike information criterion (AIC) [21] for each of these models. Prediction accuracy was estimated using the area under the receiver operating characteristic curve (AUC) as implemented in the pROC package [22] in R. Differences in AUC among models were compared by examining the change in AUC (ΔAUC) and assessed using the DeLong test [23] to determine statistical significance.

## Results

### Conditional/Joint analysis

We identified novel genome-wide significant (p<5×10^−8^) SNPs in the C/J analysis at the three following loci: *TCF7L2*, *CDKN2A/B*, and *KCNQ1* (see Table 1). In the *TCF7L2* region, we identified three SNPs (rs7917983, rs17747324, rs12266632) within a 32 kb region. The lead SNP (rs4506565) was not selected in this model, but is positioned in this region and is in moderate LD with each of the novel findings (r^2^ between 0.18 and 0.70). For each of these novel findings, the marginal effect sizes and p-values in the meta-analysis are similar to those estimated in the C/J analysis. By relaxing the p-value threshold to p<5×10^−4^, we discovered an additional SNP in this region (rs10128255). In the *CDKN2A/B* region, the lead SNP (rs2383208) was not selected in the C/J analysis. Instead, two SNPs (rs10757282 and rs10811661) approximately 1.9 kb downstream of the lead SNP were discovered. These SNPs are only 110 bases apart. rs10757282 is in relatively low LD with the lead SNP (r^2^ = 0.29). However, rs10811661 is in high LD with the lead SNP (r^2^ = 0.94). It should be noted that the correlation between the respective risk alleles is negative (r = −0.54), suggesting that estimates obtained through the single marker association were underestimated for both SNPs, as evidenced by the larger effect sizes and lower p-values estimated in the C/J analysis compared to the meta-analysis marginal association results (see Table 1). In the *KCNQ1* region, we identified two SNPs in the C/J analysis (p<5×10^−8^). One SNP (rs462402) is in moderate LD with the lead SNP, rs231362 (r^2^ = 0.45). The other SNP (rs163177) is approximately 121 kb upstream and is not in LD with the lead SNP (r^2^<0.01). By relaxing the p-value threshold to p<5×10^−6^, we identified additional novel discoveries in the *DGKB* and *TP53INP1* genes. Continuing to relax this threshold, we identify 17 (p<5×10^−5^) (see Table 1) and 34 (p<5×10^−4^) regions with multiple associated SNPs. According to PolyPhen, none of the SNPs identified through C/J analysis had any predicted functional effect. According to our query in RegulomeDB, rs387769 near *HNF4A* shows evidence of being linked to expression of a gene target, affecting binding of a transcription factor, and shows evidence of a DNase footprint. SNP rs7176681 near *ZFAND6* also displays evidence of being a transcription factor binding site, and evidence of a DNase footprint. SNP rs17168486 in *DGKB* shows evidence of transcription factor binding and a DNase peak. Several other SNPs show evidence of transcription factor binding or a DNase peak (see Table 1).

**Table 1:** SNPs identified by conditional/joint analysis with p-value <5×10^−5^, and corresponding evidence of regulatory function from RegulomeDB.

Gene region	Chr	SNP	bp	refA	freq	b	se	p	n	bJ	bJ_se	pJ	LD (r)	RegulomeDB score
*BCL11A*	2	rs2192512	59854224	C	0.475	0.058	0.014	6.50E-04	110517	0.060	0.014	2.75E-05	−0.017	
	2	rs243019	60439310	C	0.451	0.086	0.014	2.70E-06	117602	0.087	0.014	3.24E-10	0.000	5
*IGF2BP2*	3	rs12233623	186712890	C	0.548	0.058	0.014	1.00E-03	111251	0.065	0.014	5.49E-06	−0.074	
	3	rs6767484	187003272	G	0.305	0.122	0.018	3.90E-10	83684.8	0.127	0.018	7.89E-13	0.000	5
*ANKRD55*	5	rs9686661	55897543	T	0.189	0.095	0.023	9.20E-05	70923.6	0.100	0.023	1.05E-05	−0.063	4
	5	rs1895452	56096152	T	0.635	0.058	0.014	3.10E-03	118858	0.061	0.014	1.69E-05	0.000	
*ZBED3*	5	rs12522618	75491695	G	0.425	0.058	0.014	3.70E-03	112754	0.058	0.014	4.99E-05	0.009	
	5	rs7708285	76461623	G	0.300	0.122	0.022	1.10E-06	54523.4	0.122	0.022	3.70E-08	0.000	
*DGKB*	7	rs10282101	13867616	T	0.501	0.077	0.019	7.30E-05	64914.8	0.077	0.019	3.27E-05	−0.011	
	7	rs17168486	14864807	T	0.182	0.122	0.022	6.90E-07	76812	0.126	0.022	1.35E-08	−0.027	3a
	7	rs1974620	15031992	T	0.531	0.068	0.014	1.00E-04	112723	0.069	0.014	1.06E-06	0.000	
*TP53INP1*	8	rs4735337	96042641	T	0.490	0.077	0.014	6.90E-05	114412	0.078	0.014	2.01E-08	−0.022	5
	8	rs6991742	96533562	T	0.596	0.068	0.014	1.80E-04	116576	0.069	0.014	8.77E-07	0.000	5
*CDKN2A/B*	9	rs564398	22019547	T	0.579	0.077	0.014	1.70E-05	117268	0.059	0.014	2.48E-05	0.102	
	9	rs10757282	22123984	C	0.430	0.068	0.019	8.80E-04	65000.9	0.163	0.021	1.92E-14	−0.536	5
	9	rs10811661	22124094	T	0.825	0.166	0.021	1.50E-13	86410.9	0.247	0.024	1.08E-24	0.000	
*ZMIZ1*	10	rs3915932	80611942	G	0.592	0.095	0.018	4.70E-08	69629.2	0.098	0.018	7.10E-08	−0.040	5
	10	rs6480947	80906216	G	0.156	0.113	0.027	2.20E-04	59743.8	0.119	0.027	8.58E-06	0.000	
*TCF7L2*	10	rs7917983	114722872	C	0.479	0.148	0.013	1.50E-17	131838	0.082	0.014	2.17E-09	0.344	
	10	rs17747324	114742493	C	0.237	0.358	0.021	8.50E-55	70086.8	0.341	0.022	3.07E-54	−0.126	
	10	rs12266632	114754949	G	0.063	0.255	0.042	8.50E-11	53996.4	0.290	0.043	1.12E-11	0.000	5
*KCNQ1*	11	rs462402	2673869	C	0.507	0.077	0.014	1.10E-05	114391	0.076	0.014	6.49E-08	−0.002	
	11	rs163177	2794989	C	0.506	0.077	0.014	4.80E-05	114382	0.075	0.014	9.55E-08	0.040	5
	11	rs451041	3017301	G	0.496	0.068	0.014	8.20E-05	112303	0.063	0.014	8.89E-06	0.000	5
*KCNJ11*	11	rs7928810	17329019	C	0.396	0.068	0.014	1.30E-04	117423	0.068	0.014	1.50E-06	−0.004	
	11	rs757984	17576484	T	0.803	0.077	0.019	4.90E-04	102511	0.077	0.019	3.09E-05	0.000	
*MTNR1B*	11	rs10830962	92338075	G	0.408	0.104	0.014	1.50E-08	124966	0.101	0.014	1.61E-13	0.069	
	11	rs531573	92444531	C	0.182	0.086	0.018	1.70E-04	111150	0.077	0.018	2.75E-05	0.000	
*TSPAN8*	12	rs11178531	69694957	A	0.449	0.077	0.014	9.20E-06	115565	0.059	0.014	4.73E-05	0.271	
	12	rs1533104	69942814	T	0.338	0.086	0.014	2.80E-06	130115	0.072	0.014	4.26E-07	0.000	
*SPRY2*	13	rs1616547	78884037	C	0.512	0.058	0.014	3.00E-03	110308	0.058	0.014	4.84E-05	0.006	
	13	rs1327316	79607064	G	0.717	0.095	0.018	9.40E-07	82902.8	0.095	0.018	1.92E-07	0.000	
*C2CD4A*	15	rs6494307	60181982	C	0.572	0.077	0.014	1.80E-05	116797	0.077	0.014	3.65E-08	−0.001	
	15	rs2456936	60502334	C	0.785	0.077	0.019	1.10E-03	96195.2	0.077	0.019	3.32E-05	0.000	
*ZFAND6*	15	rs7176681	77714599	C	0.737	0.086	0.018	1.20E-04	85321.9	0.092	0.018	5.42E-07	−0.010	2a
	15	rs1357335	78155918	A	0.684	0.086	0.018	1.60E-05	76497.6	0.086	0.018	2.92E-06	−0.006	5
	15	rs3848174	78531707	T	0.380	0.058	0.014	3.80E-03	117013	0.061	0.014	1.78E-05	0.000	5
*HNF4A*	20	rs387769	41745269	C	0.881	0.113	0.027	1.40E-05	75082.1	0.113	0.027	2.34E-05	0.008	1f
	20	rs6073708	43386291	A	0.562	0.058	0.014	2.40E-03	111949	0.058	0.014	4.81E-05	0.000	5

Abbreviations: Chr: chromosome, bp: base pair position, refA: reference allele, freq: frequency of the risk allele, b: regression coefficient from meta-analysis summary statistics, se: standard error from meta-analysis summary statistics, p: p-value from meta-analysis summary statistics, n: sample size in meta-analysis, bJ: regression coefficient estimated from conditional/joint analysis, bJ_se: standard error estimated from conditional/joint analysis, pJ: p-value from estimated from conditional/joint analysis, LD (r): linkage disequilibrium between corresponding SNP and the following SNP at the same locus.

(1f: eQTL+transcription factor (TF) binding/DNase peak; 2a: TF binding+matched TF motif+matched DNase Footprint+DNase peak; 3a: TF binding+any motif+DNase peak; 5: TF binding or DNase peak).

### Validation/Prediction

**Figure 1:**
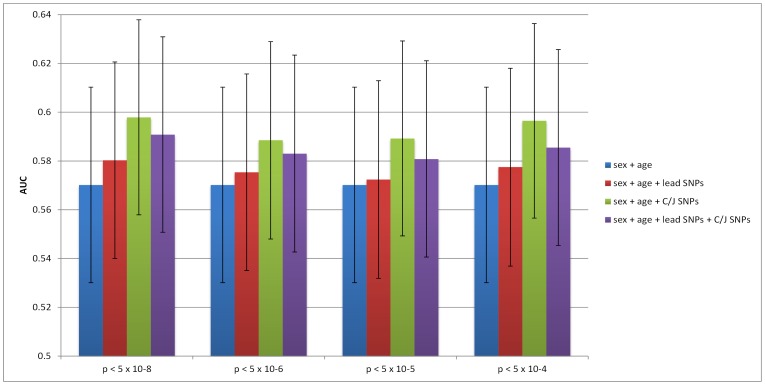
Prediction accuracy in MESA at 3 loci with additional detected SNPs at the 5×10^−8^ threshold.

The AUC of the baseline prediction model which included only sex and age was 0.5702. For each of the three loci with additional SNPs that were significant at the p<5×10^−8^ threshold (*TCF7L2*, *CDKN2A/B*, and *KCNQ1*), the inclusion of the SNPs identified by the C/J analysis resulted in a higher AUC than a model including only the lead SNP (although not statistically significant) in all regions except for *KCNQ1* (see Figure 1).

**Figure 2:**
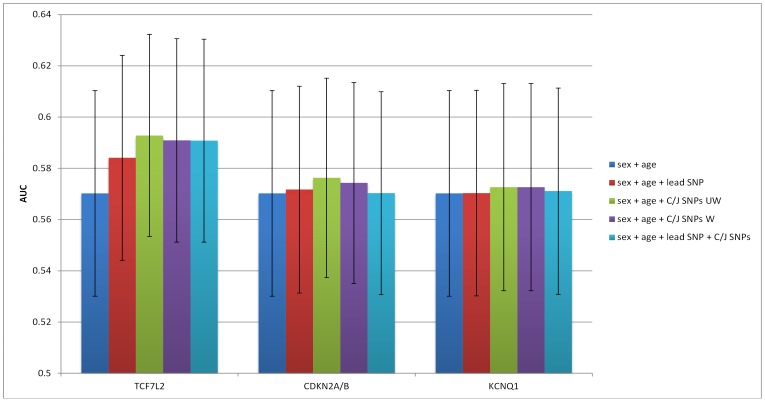
Prediction accuracy in MESA using lead SNPs vs. SNPs identified in C/J analysis at different p-value thresholds.

Considering all three loci with additional SNPs at the p<5×10^−8^ threshold collectively, we found that the use of the seven SNPs identified by the C/J analysis resulted in a slightly higher AUC (0.5979) than when using only the three lead SNPs (0.5803). This represents a doubling in ΔAUC over the age+ sex model (see Figure 2), although this difference is not quite statistically significant (p = 0.055), according to the DeLong test. The inclusion of all SNPs (lead and from C/J analysis) results in a statistically significant (p = 0.049), yet small, increase in AUC (see Figure 2). At the p<5×10^−6^ threshold, the use of 11 SNPs at 5 loci (*TCF7L2*, *CDKN2A/B*, *KCNQ1*, *DGKB* and *TP53INP1*), slightly, but not significantly, increased prediction accuracy (AUC = 0.5885) over a model considering only the corresponding 5 lead SNPs (AUC = 0.5779; p = 0.126). At the p<5×10^−5^ threshold, we observe a small increase in prediction accuracy when using the 39 SNPs identified by the C/J analysis instead of the corresponding 17 lead SNPs (AUC = 0.5892 vs. 0.5724; p = 0.079). Finally, at the p<5×10^−4^ threshold, the use of 120 SNPs identified by the C/J analysis and the lead SNPs results in a slightly higher and nearly statistically significant increase in AUC over that of a model which includes only the 34 lead SNPs at the corresponding loci (AUC = 0.5965 vs. 0.5858; p = 0.067).

Table 2 shows the proportion of variance explained by the additional SNPs identified by the C/J analysis. For each of the three loci, the SNPs identified by the C/J analysis explained slightly more of the variance in T2D risk than the lead SNP. Similarly, for the collection of SNPs identified by the C/J analysis at various p-value thresholds, we observe an increase in the proportion of T2D variance explained by the SNPs and the GRSs, along with decreasing AIC values.

**Table 2:** Variance explained at various p-value thresholds in the MESA validation dataset by the collection of individual SNPs on the liability scale, variance explained by, and model fit of, the weighted GRS, using Nagelkerke's R^2^, and AIC, respectively.

		Liability-scale variance	Nagelkerke R^2^	AIC
**age+sex**			0.0115	1448.1
***TCF7L2***	1 Lead SNP	0.0097	0.0178	1443.3
	3 C/J SNPs	0.0189	0.0212	1439.7
	4 Lead+C/J SNPs	0.0282	0.0198	1441.1
***CDKN2A/B***	1 Lead SNP	0.0004	0.0118	1449.7
	2 C/J SNPs	0.0025	0.0127	1448.8
	2 Lead+C/J SNPs	0.0029	0.0117	1449.8
***KCNQ1***	1 Lead SNP	3.00E-5	0.0115	1450
	2 C/J SNPs	0.0029	0.0122	1449.3
	3 Lead+C/J SNPs	0.0014	0.0118	1449.8
**<5.00E-08**	3 Lead SNPs	0.0101	0.0153	1446
	7 GCTA SNPs	0.0225	0.0230	1437.8
	10 Lead+C/J SNPs	0.0322	0.0197	1441.2
**<5.00E-06**	5 Lead SNPs	0.0130	0.0134	1448.1
	11 GCTA SNPs	0.0258	0.0190	1442.1
	16 Lead+C/J SNPs	0.0381	0.0164	1444.8
**<5.00E-05**	17 Lead SNPs	0.0277	0.0134	1448
	39 GCTA SNPs	0.0648	0.0209	1440
	55 Lead+C/J SNPs	0.0865	0.0171	1444.1
**<5.00E-04**	34 Lead SNPs	0.0613	0.0158	1445.5
	91 GCTA SNPs	0.1443	0.0259	1434.6
	119 Lead+C/J SNPs	0.1801	0.0197	1441.2

## Discussion

Our analyses confirm previous findings regarding the allelic heterogeneity present at the *CDKN2A/B*, *KCNQ1*, *DGKB*, and *MC4R* loci. We provide novel evidence of allelic heterogeneity at genome-wide significance at the *TCF7L2* locus. We support our finding in *TCF7L2* by showing that the use of the three identified SNPs results in a small increase in AUC (albeit not statistically significant) compared to using the lead *TCF7L2* SNP (rs4506565) alone. We observe similar but much weaker trends at the *CDKN2A/B* and *KCNQ1* loci.

At less stringent p-value thresholds, we observe additional putatively associated SNPs at up to 34 loci. Considering the collective set of loci in which additional associated SNPs were identified through C/J analysis, prediction accuracy appears to slightly improve with the addition of these additional SNPs in our validation dataset. At all p-value thresholds, the ΔAUC over the sex + age model is at least two-fold greater when using the C/J identified SNPs compared to using the lead SNPs alone.

The strength of the method developed by Yang et al. is well exemplified by the multiple associated SNPs identified at the *TCF7L2* locus, since the use of the three SNPs (which do not include the lead SNP) appears to be more informative than only using the lead SNP, rs4506565. Another example of the strength of this method is the case in which two risk alleles are in negative LD. Without the C/J analysis, the additional SNPs in the *CDKN2A/B* region would not be identified when analyzed on their own.

The main limitation of this method is that associations are not tested directly, but rather through knowledge of marginal associations, and LD patterns in a different dataset (of the same ancestral background). A major limitation of the validation stage of our study is the relatively small sample size which limits the statistical power to detect differences in prediction accuracy between different GRSs. From this perspective, it will be important to continue validating these findings in larger datasets, and to combine actual genotype data across multiple datasets instead of using summary statistics. Furthermore, it will be important to dissect the allelic heterogeneity on a locus-by-locus basis to closely examine the patterns/existence of dependencies and additive or interactive effects. Finally, it will be important to functionally characterize these as well as all GWAS findings to more firmly establish causality and better understand molecular mechanisms leading to T2D.

Nevertheless, this approach is clearly promising for a greater understanding of the molecular basis of type-2 diabetes, and potentially for use in risk prediction scores. As additional loci are identified through GWAS, it will be important to systematically identify instances of allelic heterogeneity and to examine the extent to which additional SNPs can help to shed light on the functional basis of genetic variation.
